# A new species of *Catapiestus* Perty, 1831 from China (Coleoptera, Tenebrionidae, Cnodalonini)

**DOI:** 10.3897/zookeys.809.31162

**Published:** 2018-12-19

**Authors:** Shulin Yang, Juan Guo

**Affiliations:** 1 School of Life Sciences, Guizhou Normal University, Guiyang, Guizhou China Guizhou Normal University Guiyang China; 2 Research Center for Karst Caves, Guizhou Normal University, Guiyang, Guizhou China Guizhou Normal University Guiyang China

**Keywords:** darkling beetle, Guizhou, taxonomy, southwest China

## Abstract

A new species of the genus *Catapiestus* Perty, 1831 (Coleoptera, Tenebrionidae, Cnodalonini), *C.bispinosus*, is described from Leigonsan National Nature Reserve, Leishan County, Guizhou, China. The identification key by Lang and Ren for the species of *Catapiestus* is modified.

## Introduction

*Catapiestus* Perty, 1831 is a genus in the tribe Cnodalonini (Coleoptera, Tenebrionidae), with twelve species recorded from south and southeast Asia and China; only four species were previously known from China. The species have quite uniform strongly flattened body form and coloration, and scarce or no apparent external sexual dimorphism (Lang and Ren 2009). Morphological differentiation of the species mainly depends on features of the pronotum (e.g., shape, lateral serration pattern, presence or absence of mid-longitudinal groove) and number of teeth or denticles of profemora (Lang and Ren 2009). A new species of the genus is described herein based on specimens collected from the Leigongshan National Nature Reserve, Leishan County, Guizhou, China.

## Materials and methods

Specimens were collected with six level Lindgren funnel traps which used ethanol as the lure and glycol as the killing and preserving agent in the collection bottles. Specimens were glued on pinned paper points. Labels were handwritten in Chinese. The type material is preserved in the School of Life Sciences, GZNULS (**GZNULS**). An AmScope SM-4TZ stereo microscope was used for specimen observation and dissection. Photographs were taken with a Canon EOS 6D digital camera with EOS MP-E 65 lenses.

## Taxonomy

### Catapiestus bispinosus sp. n.

Taxon classificationAnimaliaColeopteraTenebrionidae

http://zoobank.org/F117D549-B08A-4746-A05A-68EB6ED7B46A

Figures 1 , 2 , 3

#### Type locality.

*Holotype*, ♂, China, Guizhou Province, Leishan County, Leigongshan National Nature Reserve, 26°22'25"N, 108°11'58"E, 23.VI.2017, border of broad-leaf forest and Chinese white pine (*Pinusarmandii* Franch) forest, leg. S. Yang. *Paratypes*, 1♂, China, Guizhou Province, Leishan County, Leigongshan National Nature Reserve, 26°22'29"N, 108°11'54"E, 19.VIII.2011, broad-leaf forest, leg. S. Yang; 1♀, China, Guizhou Province, Leishan County, Leigongshan National Nature Reserve, 26°22'25"N, 108°11'58"E, 2.VIII.2017, border of broad-leaf forest and Chinese white pine (*Pinusarmandii* Franch) forest, leg. Yaokui Yang and Gugangzu Yang.

#### Type specimens.

***Holotype***, ♂, glued on pinned paper point, with genitalia in a separate microvial. Original label (slash, “/”, represents new line): “中国 贵州 雷山/雷公山 八公里入口处 下一/26°22'25"N, 108°11'58"E / 2017.VI.23 / 杨书林采 [handwritten label]” (translation: China, Guizhou, Leishan / Leigongshan, Entrance at 8km Lower #1 / 23.VI.2017 / leg. Shulin Yang), “HOLOTYPE: / *Catapiestusbispinosus* Yang & Guo / ♂ [handwritten on red label]”. ***Paratypes***, 1♂, glued on pinned paper point, with genitalia in a separate microvial. Original label (slash, “/”, represents new line): “中国 贵州 雷山 / 雷公山 生态定位点 / 26°22'29"N, 108°11'54"E / 2011.VIII.19 / 杨书林采 [handwritten label]” (translation: China, Guizhou, Leishan / Leigongshan Eco-monitoring site / 19.VIII.2011 / leg. Shulin Yang), “PARATYPE:/ *Catapiestusbispinosus* Yang & Guo / ♂ [handwritten on red label]”. 1♀, glued on pinned paper point. Original label (slash, “/”, represents new line): “中国 贵州 雷山/雷公山 八公里入口处 下一 / 26°22'25"N, 108°11'58"E / 2017.VIII.2 / 杨耀奎 杨光祖 采 [handwritten label]” (translation: China, Guizhou, Leishan /Leigongshan, Entrance at 8km Lower #1 / 26°22'25"N, 108°11'58"E / 2.VIII.2017 / leg. Yaokui Yang and Gugangzu Yang), “PARATYPE:/ *Catapiestusbispinosus* Yang & Guo / ♀ [handwritten on red label]”.Type specimens deposited in School of Life Sciences, Guizhou Normal University (GZNULS).

#### Differential Diagnosis.

**Figure 1.**
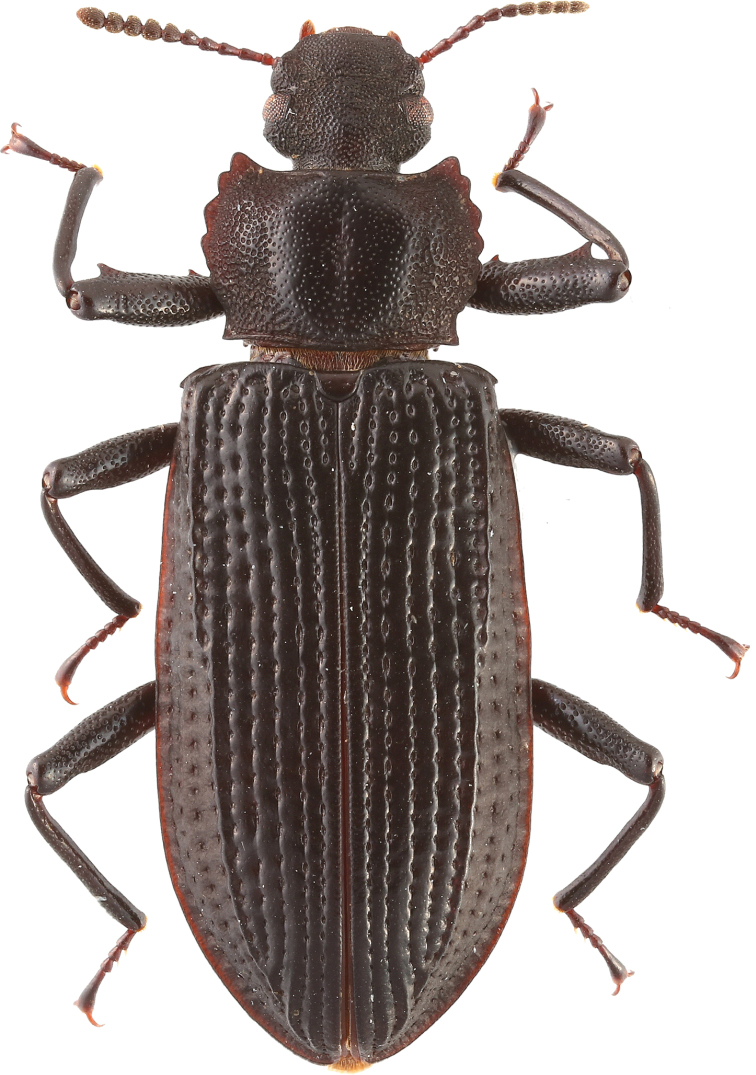
Habitus of *C.bispinosus*, male, dorsal view.

**Figure 2.**
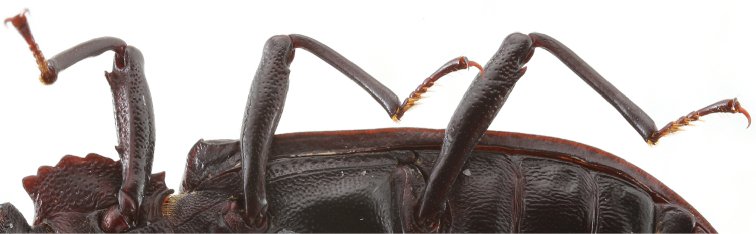
Femora of *C.bispinosus*, male, ventral view.

The new species *C.bispinosus* sp. n. has two distinctive teeth on profemora. *Catapiestusclavipes* Lang & Ren, 2009, which also has two teeth on profemora, differs from *C.bispinosus* sp. n by larger size and different shape, and tooth positions of profemora. The profemora of *C.bispinosus* are nearly parallel-sided, not expanded at base, the larger tooth is situated at apex, and a small tooth is located at the base of profemora (Figs 1, 2). The profemora of *C.clavipes* are expanded at base with a large tooth at the base and a small tooth at apex. Aedeagus of *C.bispinosus* is more rounded in lateral view and gradually widening from apex to base while aedeagus of *C.clavipes* is nearly parallel-sided in basal half of parameres and phallobase.

#### Description.

Male (Fig. 1): Body broad, flat, length 12.98–13.14 mm, width 4.02–4.1 mm. Integument dark brown to black. Head, pronotum, and elytra densely punctured, ventral head and pro-thorax, and abdomen weakly punctured and wrinkled.

**Head**: broad, trapezoidal, constricted at base to cylindrical neck. *Labrum* transverse, densely punctured and with sparsely short hairs, margin nearly rounded, with dense hairs. *Clypeus* broad, frontal slightly concave, lateral corners rounded. Outer edges of *gena* raised at antennal insertions. Fronto-clypeal suture visible as a lineal ridge, fronto-genal sulcus indistinct. Interocular space 4× eye width. *Antennae* not reaching beyond middle of pronotum when extended backwards, pedicel and 3–5^th^ antennomeres nearly conical, 6–11^th^ antennomeres clavate with shape transition from nearly triangular of the 6^th^ to oval of the last antennomere.

**Thorax**: *pronotum* transverse, raised, and with mid-longitudinal sulcus, glossy with sparse small shallow punctures in inner half, outer half inclined toward lateral margins with dense large deep punctures; anterior margin slightly concave; lateral margins arched, widest nearly at middle, serrate with 5 to 6 blunt teeth; base nearly straight with narrow ridge. *Propleura* densely punctured, pro-sternal process also punctured, gradually widening, trapezoidal, base angles acute, mesepisterna and mesepimera with denser but shallower punctures. *Scutellum* nearly semicircular, with sparse small shallow punctures.

**Elytra** nearly parallel; *epipleura* reaching apex, not glossy as other part; scutellary striole short, with 4–6 punctures; each elytron with nine punctured striae, 1^st^ and 2^nd^, 3^rd^ and 4^th^, 5^th^ and 6^th^ connected at base, respectively; intervals between 5^th^ and 6^th^, and 6^th^ and 7^th^ striae carinate, carina of interval between 6^th^ and 7^th^ striae starting at elytral base and ending at basal 2/5 of elytral length, where carina of interval between 5^th^ and 6^th^ striae starting. The two carinae connected, sometimes weakly, at basal 2/5 of elytra, forming a longitudinal plica on elytron.

**Legs**: slender with dense punctures; anterior side of ventral profemur slightly extended and ridged with two teeth, one smaller near basal third and one larger near apex. Meso- and metafemora with only one tooth on ventral anterior side of each femur near apex, larger on metafemura (Figs 1, 2).

**Abdomen**: densely and coarsely punctured, first three ventrites with longitudinal winding wrinkles.

**Figure 3.**
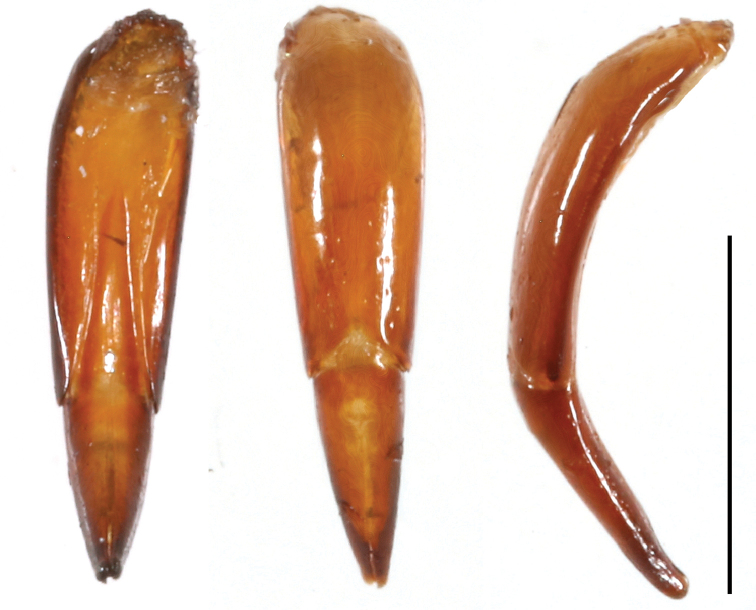
Aedeagus of *C.bispinosus*, male, left to right: ventral view, dorsal view and lateral view. Scale bar: 1 mm.

**Male genitalia**: aedeagus nearly spindle shaped in dorsal view, arcuate in lateral view, ratio of width to length 1:5, ratio of parameres to phallobase nearly 3:5 (Fig. 3).

Female, no apparent external sexual dimorphism except body slightly smaller, length 12.66 mm, width 3.74 mm.

#### Etymology.

The name of the new species refers to two spine-like teeth on each profemur.

#### Distribution.

China: Guizhou, Leishan, Leigongshan.

#### Discussion.

The range of *Catapiestus* in China has a distributional gap between Yunnan and Fujian provinces below 30°N in southern China. The discovery of *C.bispinosus* in Guizhou province presents a range extension for the genus and a provision for new species and distribution records of the genus in the area of southern China between Guizhou and Fujian provinces.

##### Modified couplets to the key to *Catapiestus* by Lang and Ren (2009)

The couplets 2 and 3 of the key to *Catapiestus* by Lang and Ren (2009) should be modified as follows to receive *C.bispinosus* sp. n.

**Table:** 

2	Profemur with 2 teeth	**2a**
–	Profemur with 1 or 3 teeth or denticles	**3**
2a	Profemur strongly expanded at base, with 1 large tooth at widest point, 1 small tooth at apex	*** C. clavipes ***
–	Profemur not expanded at base, nearly parallel-sided, with 1 small tooth at base and 1 large tooth at apex	*** C. bispinosus ***
3	Profemur with only 1 tooth in front margin	**5**
–	Profemur with 3 denticles	*** C. mediocris ***

## Supplementary Material

XML Treatment for Catapiestus bispinosus
